# Monitoring and risk assessment of airborne graphene oxide released during a pilot study of 3D printing with polylactic acid filaments

**DOI:** 10.1007/s40201-026-00998-w

**Published:** 2026-08-07

**Authors:** Ciro Salcines, Maidá Domat, Javier Ruiz, Laura Castanon-Jano, Elena Blanco-Fernandez, Rafael Valiente

**Affiliations:** 1 Health and Safety Unit, Universidad de Cantabria, Santander, 39005 Spain; 2 Department of Physics, Area of Applied Physics, Universidad de Oviedo, Oviedo, 33007 Spain; 3 Area of Health and Safety, FREMAP - Mutua Colaboradora con la Seguridad Social, nº 61, Majadahonda, 28222 Spain; 4 Area of Manufacturing Processes, Universidad de Cantabria, Santander, 39005 Spain; 5 GITECO Research Group, Universidad de Cantabria, Santander, 39005 Spain; 6 Department of Applied Physics, Faculty of Sciences, Universidad de Cantabria, Santander, 39005 Spain

**Keywords:** Graphene oxide, 3D printing, Nanoparticle emissions, Occupational exposure, Risk assessment

## Abstract

Fused Deposition Modeling technique is a widely used 3D printing technique that often employs polylactic acid filaments (PLA), which can be enhanced with nanomaterials such as Graphene Oxide (GO). During thermal processing, these materials may release airborne particles, raising occupational and environmental exposure concerns. This study evaluates potential exposure to aerosolized GO from PLA filaments using a tiered risk assessment approach based on the European standard EN 17058:2018 and the Organization for Economic Co-operation and Development (OECD) guidance. A monitoring strategy combined near-field and far-field particle counters with Scanning Electron Microscopy (SEM) and Raman Spectroscopy, and volatile organic compounds (VOCs) were assessed to compare with Spanish and Finish 2022 threshold occupational exposure limits. Nanoparticles and their agglomerates were detected at low number concentrations on average, with traces of Graphene Oxide in respirable fractions. These findings warranted further validation using statistical models such as ARIMA. Although concentrations were below proposed limits. The presence of GO in respirable aerosol indicates that GO nanoestructured within PLA was released into the workplace air. This finding underscores the need for tailored monitoring and control measures in occupational environments.

**Supplementary Information:**

The online version contains supplementary material available at 10.1007/s40201-026-00998-w.

## Introduction

Additive manufacturing technology, more familiarly known as 3D printing, presents known advantages over conventional technologies, such as producing customized molds. This method helps to manufacture on demand with shorter supply deadlines. Also, additive manufacturing presents environmental advantages with respect to subtractive fabrication, because the material used is minimized by reducing the waste generated during the manufacture [1, 2]. Due to the reduction of the price of some commercial printers, especially for polymers, the fabrication costs with this technique have become cheaper, being competitive in relation to other conventional techniques, like extrusion molding for plastics. In this sense, a niche market is being identified to provide a solution to customers’ requirements concerning the demand for personalized complex shapes with a limited number of units and short supply deadlines. In relation to the global sector of additive fabrication of any material and technology, this is expected to reach 4500 million USD in 2025 [3].

In the field of polymer printing, extrusion is one of the most used technologies [4]. The polymer extrusion technology was developed by Scott Crump in 1989 and was patented with the name of ‘Fused Deposition Modeling (FDM)’ [5], although it is also known as ‘Fabrication of Fused Filament (FFF)’. FDM consists of extruding a polymeric filament through a heated extruder head, depositing it layer by layer.

The most commonly used polymer used in the FDM technique is the polylactic acid (PLA) filaments [6] followed by acrylonitrile butadiene styrene (ABS) [7]. The development of new composite filaments combining a polymer and a mineral/organic powder or fiber aiming to improve certain properties, such as mechanical, electrical, insulation and optical properties, among others, is gaining major attention nowadays. Composite filaments have been used in the fields of biomedical, tissue engineering, bioprinting, electrical conductivity, automotive, aviation, sensor, battery, robotic, and smart textile industries among others [6]. One of these composites, which is now available in the 3D printing market, is the conductive PLA-Graphene Oxide (PLA + GO), which is the focus of this study. The GO nanoparticles improve mechanical strength, thermal and electrical conductivity, and thermal stability as reported elsewhere [8, 9].

However, as in many other scenarios, the potential health risks to employees and users of these 3D printers using embedded nanomaterials are often underestimated. The formation of aerosols and suspended nanomaterials during the melting process cannot be ruled out. In fact, Stefaniak et al. [10] showed release of polymer particles containing carbon nanotubes from FFF printing with PLA and other polymers.

Exposure measurements of 3D printers with different filaments (ABS, Nylon or PLA) have shown presence of nanoparticles (NPs) [11–14] along with emissions of Volatile Organic Compounds (VOCs) released from the process [13–15]. According to these studies, FDM process with PLA filament emit both NPs and VOCs, with exposure measurements at laboratory conditions reaching Particle Number Concentrations (PNC) up to 10^3^−10^5^ pt/cm^3^ [16, 17]. The necessity of offline identification of these NPs has been remarked by a study collecting samples with cassettes and characterized them using FE-SEM/EDX, confirming the NPs emission [18, 19]. Besides, the increasing availability of filaments with additives such as metals, carbon nanotubes, or graphene [20], raises further concerns.

Previous studies have also shown that graphene and GO can penetrate the respiratory system and reach the bloodstream, potentially causing adverse health effects [21]. Their capacity to produce reactive oxygen species (ROS) contributes to the potential toxicity of these NPs. Therefore, it is essential to develop measuring methodologies capable of identifying the specific nanomaterial released in the workplace. Establishing and following standard criteria should be a priority.

This research performs a comprehensive characterization of released nanoparticles using Raman Spectroscopy [22, 23] and SEM; a combination which has not been extensively covered in previous literature to date. The application of ARIMA statistical analysis ensures the best quality of data and fully describes particle dispersion. Exposure measurement by applying the European standard EN 17058:2018 [23] makes it possible to apply standardized protocols in real environments. Advanced analytical techniques enable the accurate identification of GO particles, providing evidence of a new nanomaterial exposure route effecting 3D printing workers (and potentially for any user, given the popularity of these printing tools), caused by nanostructured filament, being released during the printing and resulting in the presence of GO in the respirable aerosol.

Previous studies on PLA/graphene composites mainly focused on characterizing the printed materials by SEM or Raman [24, 25] while several emission studies reported NPs and/or VOC release during FDM without considering nanofillers embedded in the filament [13, 26]. To our knowledge, exposure assessments of airborne GO from PLA + GO during desktop FDM remains scarce. This study directly addresses this gap and provides valuable insights for assessing health risks and prevention measures. Overall, these findings contribute to more precise exposure assessments and greater reliability in evaluating safety in the rapidly evolving field of additive manufacturing.

## Materials and methodology

### Exposure scenario

The monitoring took place in a laboratory used exclusively for 3D printing, whose dimensions are 67 m^2^ and volume 206 m^3^. This workplace has neither forced ventilation, heating nor air conditioning. During the measurements, the door and windows were closed. There were no airflows which could generate positive or negative air pressure [23]. The experiment was conducted in a closed, unventilated room as a worst-case scenario. While such conditions may amplify emissions compared to some workplaces. This reflects real workplace conditions of this 3D printer and the situation found in most facilities, allowing an upper-bound estimate of potential exposure.

The 3D Printer used was an Artillery Sidewinder X1 having an open design without enclosed protective or local exhaust ventilation. The filament was PLA + GO with a 1.75 mm diameter. Wojtyla et al. [27] demonstrated the thermal degradation of PLA filament during 3D printing, so 200 °C was the lowest temperature compatible with high quality results. During the first printing process, three pieces of 10 × 60 × 250 mm, with a total weigh of 549 g were printed with a total run length of 23 h 35 min, at a rate of 0.39 g/min. In a second printing process, only one piece was printed during 3 h and 15 min with the same rate.

### EN 17058:2018 - tiered approach

The measurement strategy followed in this study was based on the European standard EN 17058:2018 and the report published by the Organisation for Economic Co-operation and Development (OEDC) [28]. This approach is divided in three stages: finding out contextual information connected with the work conditions (Tier 1), establishing the exposure at basic level (Tier 2), and confirming the exposure at detailed level (Tier 3).

This study has been based on Tier 1 and Tier 2 approaches, where the first stage consists of gathering information and the second one, stablishing the exposure with portable direct reading devices along with offline analysis of collected samples. In case the results are not conclusive, a statistical analysis of the data by means of Autoregressive Integrated Moving Average (ARIMA) can be performed to understand at a deeper level the relationship of the measured data.

The main objectives are to assess exposure by inhalation of nano-objects and their aggregates and agglomerates (NOAA) at workplace and apply the risk management measures needed.

### Direct measurements

A combination of real-time instruments was placed at different locations to track the rate of the released materials during the printing process. VOCs and particulate matter were measured at Near Field (NF), while particulate matter was only measured at Far Field (FF), although in both locations Condensation Particle Counters (CPC) were measuring simultaneously [23, 29]. Details of the instruments can be seen in Table 1.

#### Far field – background

To monitor the background PNC, a P-Trak Ultrafine Particle Counter (8525-TSI) was located 9 m away from the 3D printer. During the first printing process, measurements were taken in two stages due to the lack of night supervision of the devices. The first stage started 2 h before printing and lasted for 12 h, with a short break to change batteries. The second stage started 90 min before printing finished and lasted over 30 min. The equipment measured made measurements at intervals of 1 min.

#### Near field – breathing zone

As per standards [23], the equipment was located at 60 cm from the source. The height (120 cm) represented the breathing zone of a sitting research worker handling the 3D printer. This location corresponds with the worst-case scenario, and where the researcher’s exposure would be the highest concentration. Real-time PNCs were recorded with two devices, a water-based Condensation Particle Counter (CPC, 3786-TSI) and an Aerodynamic Particle Sizer (APS, 3321-TSI). This equipment is used to measure NOAAs [30]. During the first printing process, measurements were taken at two periods: one started 20 min before printing and recorded for 10 h, and the second one started 90 min before printing finished and continued for 144 min. The equipment measured at intervals of 1 min. Alongside the instruments; a CO_2_ monitoring device (ARANET4) was also placed.

**Table 1:** Characteristics of the particle monitoring devices

Device	Model	Location	Particle Size Range	Concentration Max. Range
CPC	3786-TSI	NF	0,025–3 μm	10^5^ pt/cm^3^
P-trak	8525-TSI	FF/BG	0,02–1 μm	5 × 10^5^ pt/cm^3^
APS	3321-TSI	NF	0,5–20 μm	10^4^ pt/cm^3^
Cascade Impactor	SKC Sioutas	NF	< 0.25–2.5 μm	
ARANET4	PRO	NF	--	0–9999 ppm CO_2_

Different instruments were used at near-field (NF) and far-field (FF) mainly due to the limited availability of monitoring devices. To ensure some degree of comparability, both CPC and P-Trak (which operate on the same detection principle) were placed at NF and FF, while the APS, cascade impactor, and CO₂ monitor were concentrated in NF to better characterize emissions directly at the source. This distribution allowed us to capture both source-related and background variations, even if instrument sensitivity ranges were not fully identical.

### Collection of samples and offline analysis

#### Particulate matter

The direct measuring equipment cannot distinguish source particles from background. The fact that GO can be dispersed in airflow makes it necessary to collect samples and characterize them with offline methodologies, such as scanning electron microscope (SEM) or transmission electron microscopy (TEM) [31, 32]. For this purpose, Spinazzè et al. [33] studied how to obtain multi-metric occupational exposure assessments of graphene family nanomaterials.

Samples were collected at NF with a SKC Sioutas cascade impactor. A Leland Legacy pump was kept at a constant flow rate of 8.5 l/min. Filters collected a total volume of around 10,000 l for the whole 3D printing process. Samples collected with the PTFE filters of the Sioutas with different sizes (0.25, 0.5, 1 and 2.5 μm) were analyzed using Raman spectroscopy and compared with a sample of black thread (PLA + GO) before printing, a printed black strip (PLA + GO) and a white filament (PLA) as control. SEM measurements on samples collected with the filters could not be done at high resolution due to sample instabilities when exposed to over 15KeV, being limited to a maximum magnification of 30000x and thus preventing clear nanoscale visualization. While SEM provided only general surface morphology, it could not definitively identify GO structures. Therefore, SEM and Raman Spectroscopy techniques were used to achieve a more reliable level of characterization of the collected nanomaterials [32]. The T64000 Horiba-Jobin-Yvon Raman spectrometer equipped with a cryogenic cooled CCD detector in substractive mode was used to analyse PLA filaments, unit printed and collected samples. Afterwards a SEM ZEISS EVO MA15 equipped with CCD INCA x-act Model:51-ADD0048 10 mm² Silicon Drift Detector (SDD) incorporated with INCA EDS Analysis Software was used to characterize the samples [32, 34].

The chemical and structural composition of the samples were studied through Raman spectroscopy. The spectral resolution of the system was about 0.6 cm^–1^. For excitation, the 514.5 nm line of an Ar^+^-Kr^+^-laser was focused on the sample using a 20X objective, while the laser power was kept below 2 mW, measured directly before the sample, to avoid laser-heating effects on the probed material and the concomitant softening of the observed Raman peaks. The phonons frequencies were obtained by fitting the experimental peaks to Lorentzian functions. This technique should provide chemical and structural analysis, including estimation of the size or thickness of GO layers [30].

To address the Material Safety Data Sheet gap information, characterization of the PLA + GO filament, PLA filament, and 3D printed pieces with PLA + GO were conducted.

#### VOCs

Aldehydes (acetaldehyde and formaldehyde), caprolactam, 1-butanol, etilbenzene, styrene and methyl methacrylate were chosen to compare with Spanish 2022 threshold occupational exposure limits [35]. For Benzaldehyde, the Finish occupational exposure limits [36] were taken as a reference.

According to the standard, the measurement was taken for 15 min at the end of the 3D printing process, with the aim of detecting the highest concentration possible, which is being the worst-case scenario [37]. Similarly to [41–45], VOCs were collected closed to the breathing zone of a sitting worker handling the 3D printer, following the recommendations for sample collection analysis [38–43].

Sample pumps inlet airflows were set as follows: for the acetaldehyde/formaldehyde/benzaldehyde, 0.2058 l/min; for caprolactam, 1.0110 l/min; for 1-butanol/etilbenzene, 0.206 l/min; for styrene, 0.2004 l/min and for methyl methacrylate, 0.049 l/min.

### Statistical analysis

In the previously collected measurements, data points might exhibit autocorrelation, meaning they are not independent. This can lead to erroneous conclusions if not accounted for. For an appropriate statistical analysis, the application of ARIMA models is proposed to have a second printing process in the standard EN 17058:2018. ARIMA models, although seldom employed in real-time nano exposure data analysis, are well-suited for analysing and predicting time series data by addressing autocorrelation. The step-by-step procedure with examples is explained in detail by Klein Entink et al. [44] therefore, it will not be repeated here. Since 12 samples of 15 min each from the printing process are analysed in a second printing process, only a summary of the outcomes of each set of measurements is presented.

## Results

The assessment strategy taken included direct reading of instruments and time-integrated offline sampling to determinate whether there was an exposure to NOAAs, since monitoring is required, and sampling is recommended in the basic assessment Tier 2 from EN 17058:2018.

### Tier 1. Initial assessment

Tier 1 involves gathering initial information to assess the potential release of NOAAs. Three options follow this assessment: confirming high potential release of NOAAs and proposing mitigation measures, leaving the situation unchanged if there is no indication of release, or conducting a Tier 2 investigation if release cannot be ruled out.

Since the potential release of incidental nanoparticles are induced by the previous literature presented and no mitigation measures are in place, we jumped to the next tier.

### Tier 2. Real-time measurements

The time series gathered by the different real-time monitoring instruments during the 3D printing are described hereafter in terms of particle size distribution, number concentration as well as mass concentration. The results presented correspond to the data collected at the NF and FF locations.

Table 2 shows the average of each instrument at the mentioned locations, along with the maximum and minimum values. In the case of the APS, the averaged measurements are shown, which are based in particle number (PNC), particle surface (PSC) and particle mass (PMC) concentrations, respectively.

**Table 2:** Summary of the average, minimum and maximum concentrations and geometric mean diameter recorded by the different instruments during first printing process

	Near Field				Far Field
	APS			CPC	*P*-Track
	PNC	PMC	PSC	(x10^3^ pt/cm³)	(x10^3^ pt/cm³)
	(pt/cm³)	(µg/m^3^)	(µm²/cm³)		
Mean	67.02	41.36	46.75	2.10	2.97
Min -Max	54.36–312.02	6.78–631.42	4.49–533.09	0.61–13.0	1.92–6.34
Std. Dev. – 95% CI	31.19–2.24	75.64–5.44	60.08–4.32	0.65–0.05	1.25–0.09
Geo. Mean Diameter (µm)	1.04	9.99	4.99	*n/a*	*n/a*
Std. Dev – 95% CI (µm)	1.81–0.13	1.94–0.14	2.66–0.19	*n/a*	*n/a*

n/a: not applicable

The relatively high standard deviations observed, particularly at the NF, reflect the strong temporal variability of particle release during the printing process rather than measurement uncertainty. Such fluctuations are consistent with previous studies on 3D printing emissions [45] and highlight the episodic nature of ultrafine particle generation. The 95% Confidence Intervals (CIs, t-based) are reported on 1-min averages to mitigate autocorrelation from continuous 1-sec measurements.

#### PNC during 3D printing

**Fig. 1:**
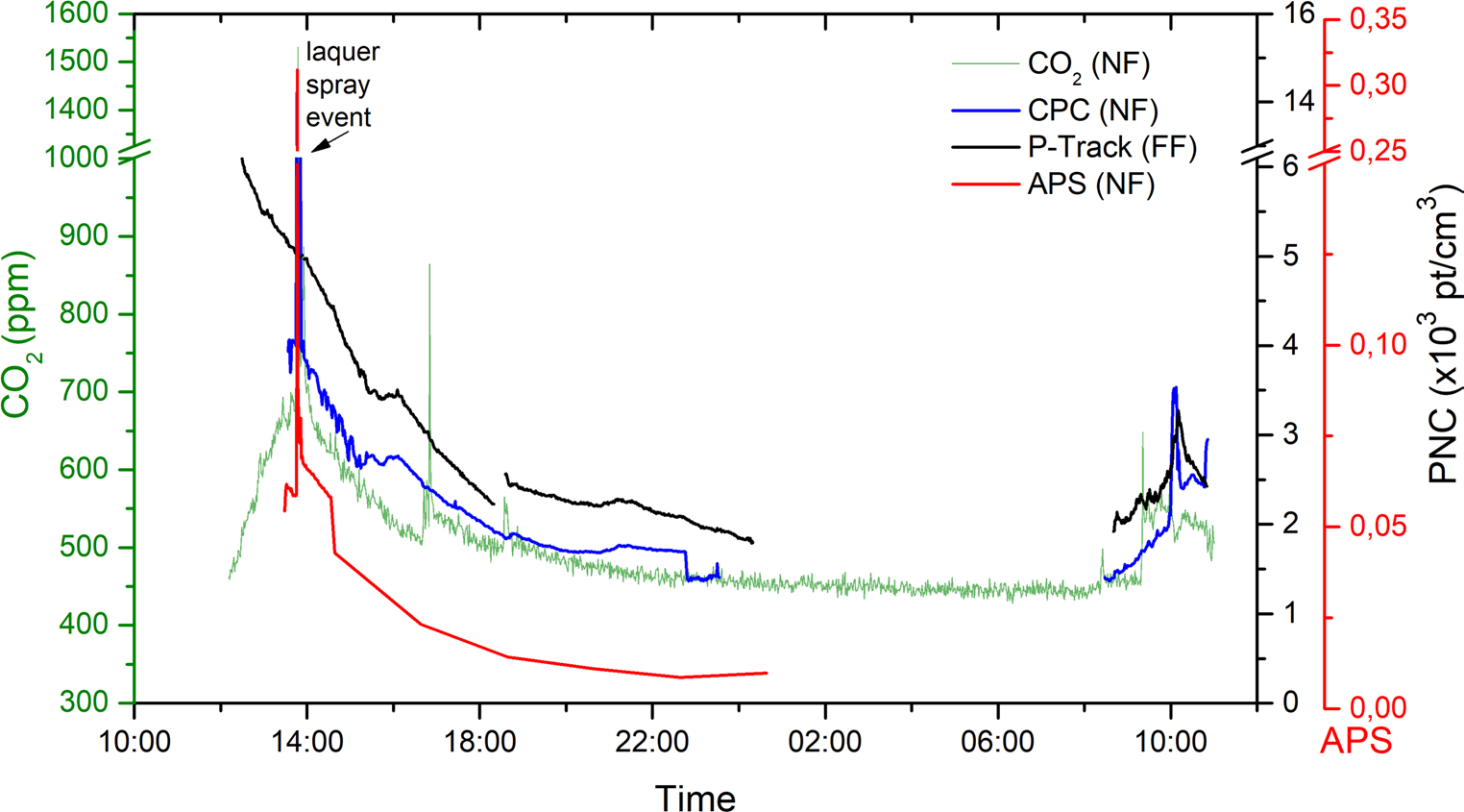
CO_2_ concentration in the NF (left axis) and PNC in NF and FF (right axis) during 3D printing

Figure 1 shows the PNC from the CPC and the APS in the NF and the P-Track in the FF, with data averaged every minute, along with the CO_2_ concentration registered with the ARANET4 PRO. In the case of the 3D printing process, CPC, P-Track and APS stopped measuring at midnight. Therefore, the x-axis and y-axis of Fig. 1 were scaled. In the case of the y-axis, representing concentration, there is a maximum for all NF instruments that masks the rest of the data. This maximum occurred during the pre-print coating to improve adhesion with lacquer spray at 13:47, being a one-off, short-range event only detectable nearby the printer and non-related with the composition of the filament.

From the trend of the data, all the instruments register a downward tendency during the whole 3D printing process, despite of being a non-encapsulated hot process without any engineering controls during operation, which would lead us to expect an increase in emissions.

The Particle Size Distribution (PSD) measured with the APS in the NF pointed out that the PNC remains rather low for particles over 2 μm and the highly populated contribution of the coating spray at the beginning, with particles below 1 μm. The geometric mean particle size, related to PNC, is 1.04 μm.

It is noteworthy that the NF values measured with the CPC are lower than the FF values measured with the P-Trak. Considering that the CPC covers a larger range of particle sizes than the P-Track (up to 3 μm and 1 μm, respectively), the contribution cannot be due to the difference in size range. Rather, it suggests that the higher PNC in the FF reflects background contributions independent from the process. At 9:57 am, second day, the 3D printing process ended, but the devices continued measuring for another 54 min. It can be seen from Fig. 1 that there is a concentration peak in both NF and FF when printer was stopped, after which PNC drops sharply.

On the other hand, the trend of the CO_2_ concentration is very similar to that of particles. Since it was started prior to the particle instruments, it shows an initial increase in the CO_2_ curve, in part due to the number of people in place setting up the instruments, and part from the heating of the equipment and the filament of the printer. This curve goes down as soon as the printer is stabilised, and the concentration continues steady for the next 24 h, increasing slightly the next morning from 9:00 am as the researchers enter the lab again.

#### Mass concentration during 3D printing

**Fig. 2:**
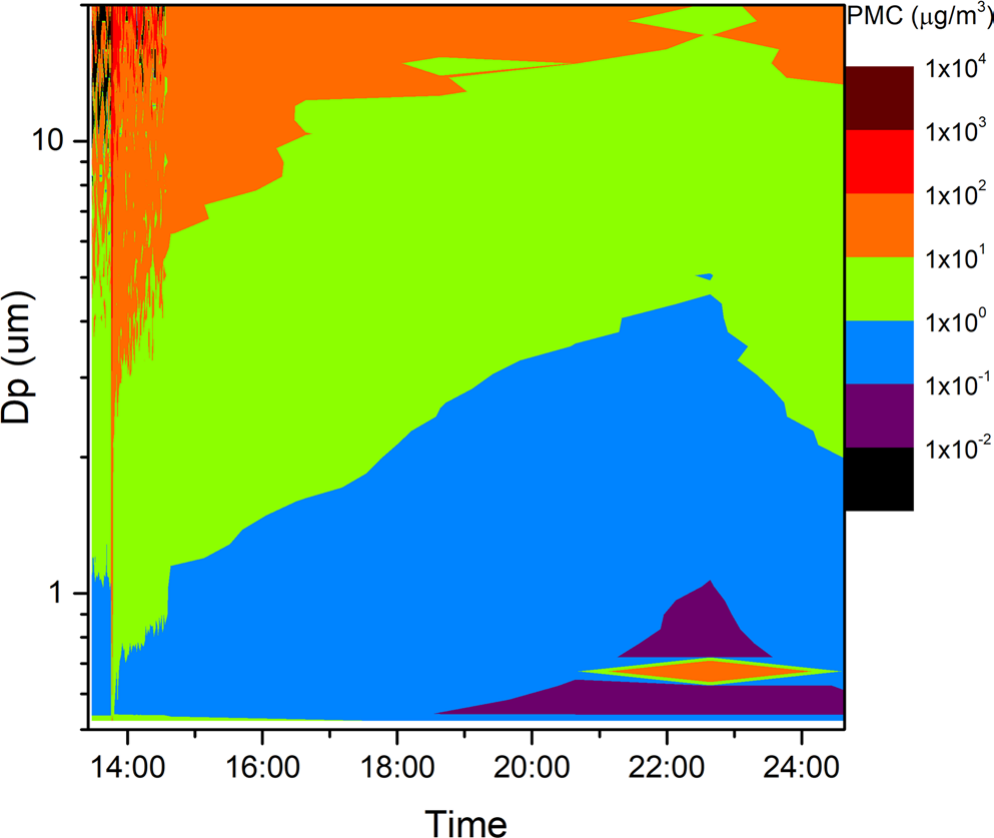
Time and particle size dependence of the PMC measured with the APS in the NF

Figure 2 shows the temporal evolution of the mass concentration. The average of the total mass of the particles is about 40 µg/m^3^ for a geometric mean diameter of 10 μm, meaning that few coarse particles were present during the 3D printing process.

#### Surface area during 3D printing

**Fig. 3:**
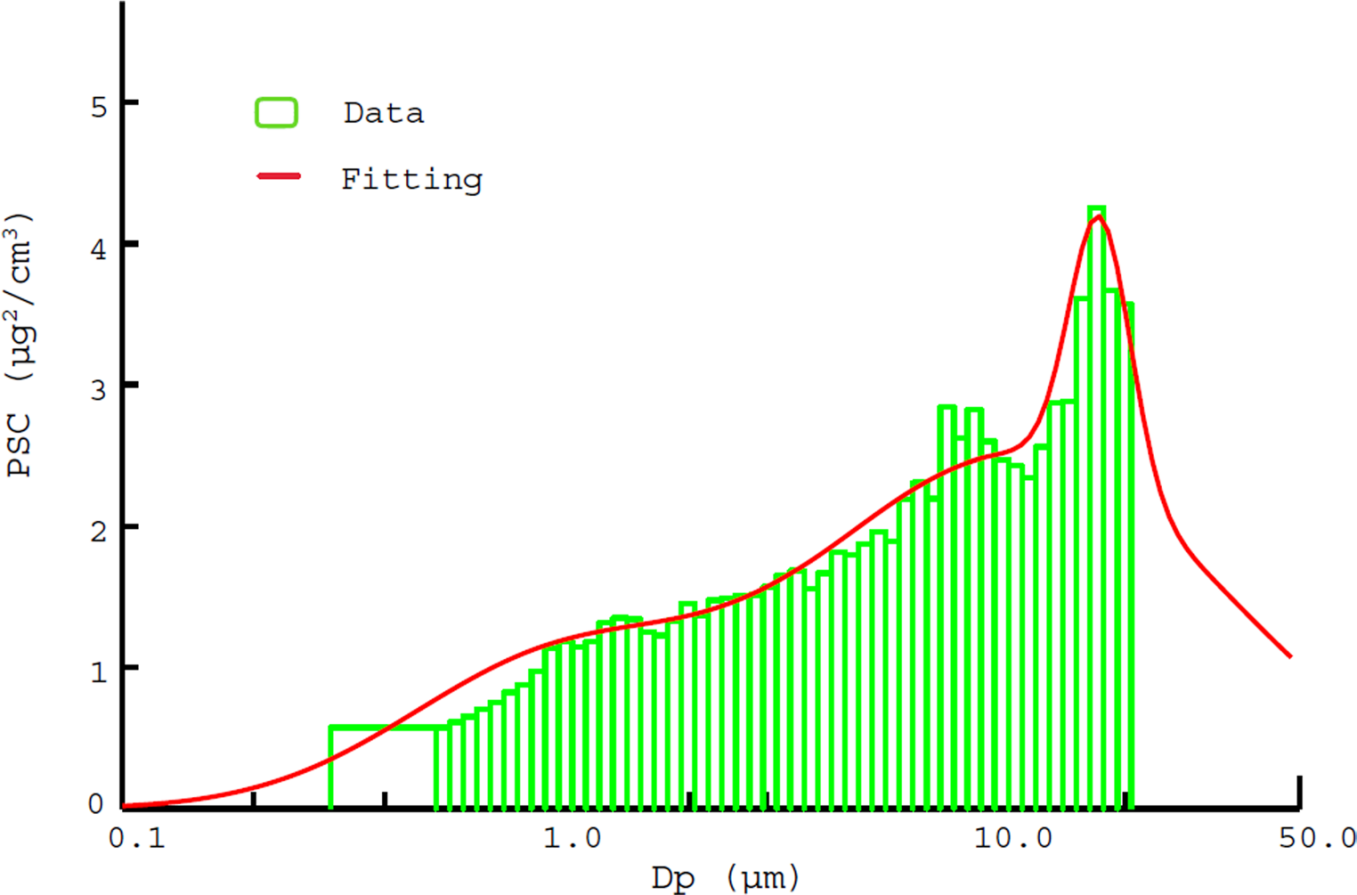
Log-normal fitting of the surface area distribution registered by the APS

The particle concentration, regarding the surface area of the particles, is a much more useful metric for exposure and toxicological assessment of carbonaceous materials [46, 47], since it can be directly related to the Lung Deposited Surface Area (LDSA). The histogram from Fig. 3 shows that the PSD by surface area (from the APS software) is a multimodal distribution. The log-normal fitting of the peaks performed as presented in Fernandez-Díaz et al. [48] suggests that at least three particle sizes at around 0.97, 11.3 and 17.4 μm (with a relative error of 8%) are detected, data shown in Table 3.

**Table 3:** Characteristics of the modes fitted from the surface area distribution from the APS

Mode nr.	PSC (µg^2^/cm^3^)	D_pg_ (µm)	Std. dev. (µm)
1	8.48	0.97	2.2
2	30.1	11.3	3.0
3	3.27	17.4	1.2

The leftmost mode, at 0.97 μm, likely reflects accumulation-mode agglomerates which can be originated from condensational growth or coagulation of ultrafine emissions from the printing process [49] while the modes at 11.3 and 17.4 μm suggests the presence of coarse and very coarse particles respectively, not only due to the emissions from the printing process, but also perhaps enhanced by side activities such as lacquer sprays and resuspension from the transit of personnel through the room [50].

Because the APS works with aerodynamic diameter, irregular and/or low-density carbonaceous agglomerates like GO behave as if larger, so the APS reports a larger apparent size, since density and shape affect the aerodynamic mobility [51].

**Fig. 4:**
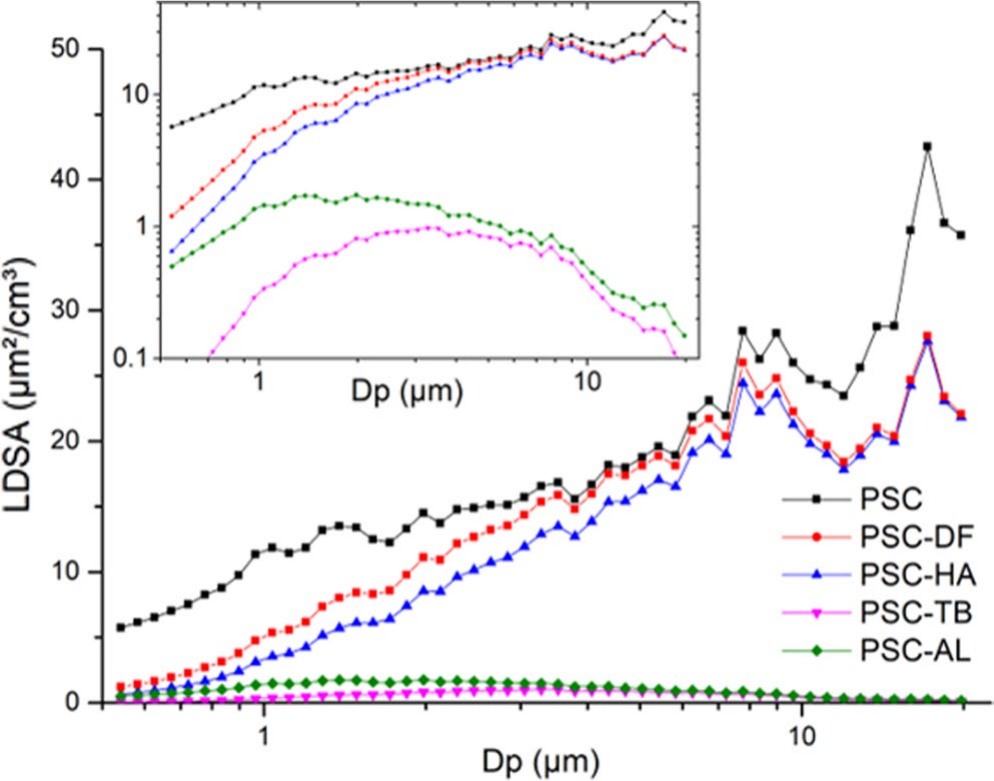
Total and deposited surface area by respiratory region based on the size corresponding to the ICRP deposition model. In the inset, the same plot but in double logarithmic scale

Since the surface area is also related to toxicological effects, it is interesting to analyse the fraction of particles as a function of the surface area lying on each part of the human respiratory tract. For this purpose, the Human Respiratory Tract Model (HRTM) from the ICRP model [52] was used. The predicted fraction of total and regional deposition of particles in the three main regions of the respiratory tract: the Head Airways (HA, where the surface of the extra-thoracic region is estimated to be 470 cm^2^), Tracheobronchial (TB, with an airway surface of the bronchial and bronchiolar region equal to 302.6 cm^2^) and Alveolar (AL, with an airway surface of the alveolar-interstitial region equal to 147.5 m^2^), scaled to the aerodynamic diameter range measured by the APS. In Fig. 4 the deposited surface area in each of the regions is estimated by multiplying the surface area concentration for each aerodynamic diameter measured by the APS times the percentage of deposition on each region provided by the HRTM. These results indicate that most of the particles are deposited in the HA, although below 2 μm there is a significant fraction deposited in the AL.

**Fig. 5:**
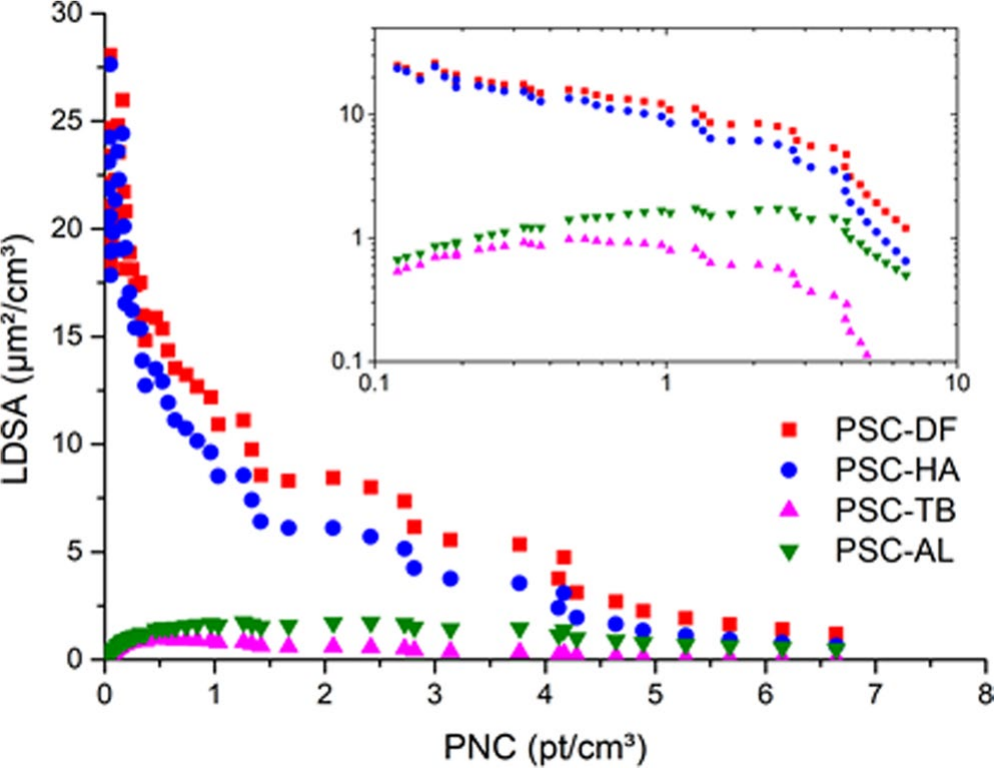
Lung Deposited Surface Area (LDSA) versus PNC for the APS aerodynamic diameters. The inset shows the same plot but in double logarithmic scale

Figure 5 presents the relationship between the LDSA and the PNC for each respiratory region. As expected, there is an inverse relationship: the most abundant particles contribute minimally to the overall surface area, and a relatively small fraction of particles with larger surface area dominates the deposition. However, this trend is not uniform across regions. The HA and AL regions receive the majority of high-surface-area particles, both with quite large probability., This suggests that, although HA works as a primary filter, there exists a non-negligible deeper lung penetration. In contrast, the intermediate TB region contributes minimally to LDSA, indicating limited particle residence in this region. The log-log inset within Fig. 5 highlights these differences. It must be noted that measuring with the APS, a portion of the sub-0.5 μm fraction is not aerodynamically sized, thus AL fraction from fine and ultrafine particles may be underestimated.

### Offline analysis

#### VOCs

VOCs detected were evaluated based on a short-term exposure approach, using a 15-minute sampling period in accordance with standard methodologies [43, 53, 54]. As no repeated measurements were conducted, the resulting data may not be fully representative of typical exposure variability and represent a worst-case snapshot rather than a full exposure assessment. Table 4 summarizes the results. In the case of exposures to sensitising, carcinogenic, mutagenic, reproductive toxic or endocrine disrupting agents, even if an acceptable exposure assessment has been obtained on the basis of the corresponding Exposure Indexes [35, 36], all reasonably feasible specific preventive measures must always be taken in order to reduce the risk to the minimum possible, since for these agents there are no “safe” exposures, even if there is an indicative Occupational Exposure Limit (OEL).

For carcinogenic or mutagenic agents of the first category, the corresponding preventive measures derived from the application of Spanish Royal Decree RD 665/1997 and European Council Directive 90/394/EEC [55, 56], and subsequent modifications must be implemented.

**Table 4:** Concentrations for single short-term exposure evaluation of the selected VOCs

Agent	H phrases^⁑^	Concentration for short-term^*^ exposure (mg/m^3^)^⁑, ⁂^		Exposure Index^⁑, ⁂^ A/B
		Sample collected (A)	Exposure limit (B)	
Caprolaptama	332-302-319–335-315 335	0.79	40^**⁑**^	0.02
Styrene	226-361d-332–372-319-315	9.65	172^**⁑**^	0.056
Ethylbenzene	225–332-373-304	1.98	154^**⁑**^	0.01
Butanol	226–302-335-315-318–336	1.65	154^**⁑**^	0.01
Methyl methacrylate	225–335-315–317	40.82	460^**⁑**^	0.09
Benzaldehyde	226–302-312–400	0.10	17.4^**⁂**^	0.01
Acetaldehyde	224–350-341-335-319	0.10	46^**⁑**^	0.002
Formaldehyde	350-341-301–311-331-314-317	0.10	0.74^**⁑**^	0.13
Additive effects				
Σ aldehydes				0.142
Styrene + Butanol + Ethylbenzene				0.076

*A single 15-minute measurement [43, 53, 54]

^⁑^ H phrases and OELs in Spain [35]

^⁂^ OELs in Finland [36]

#### SEM-EDX characterization

**Fig. 6:**
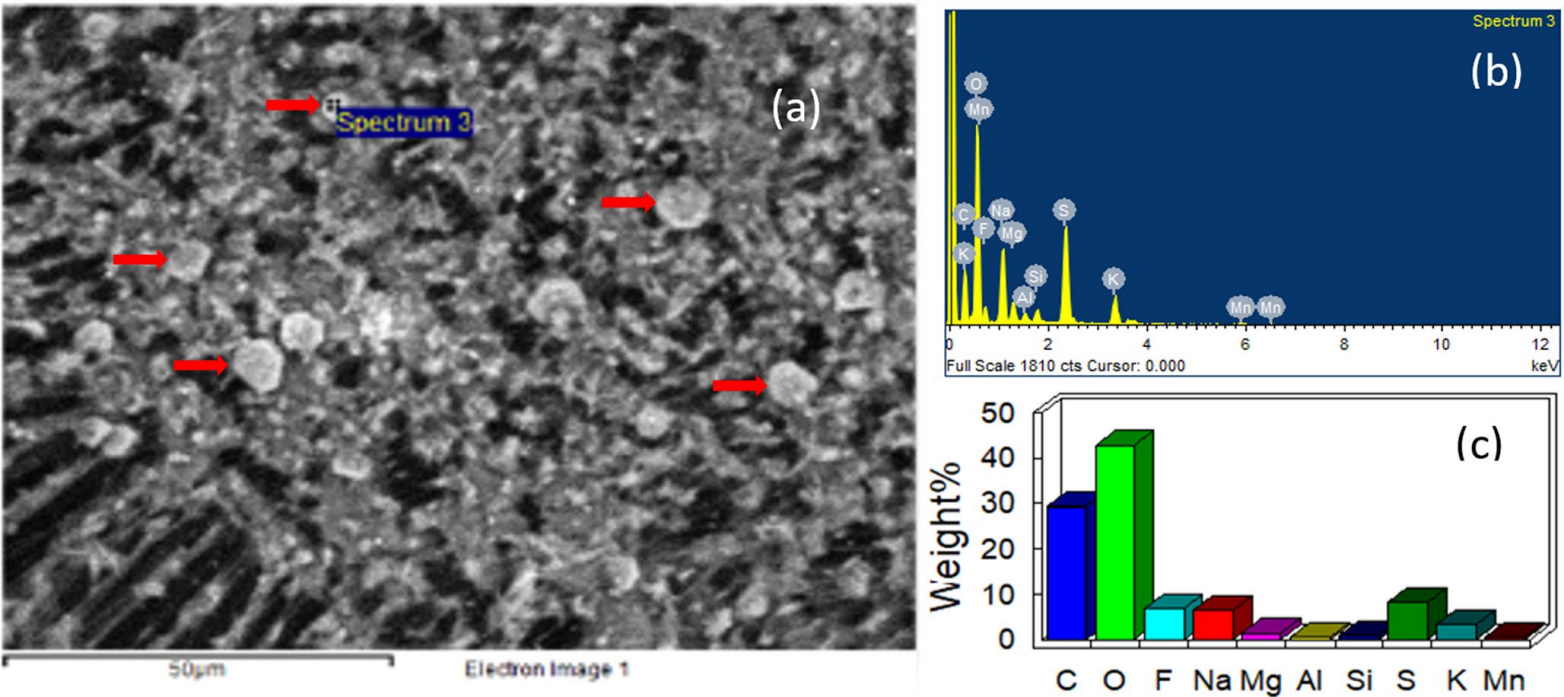
(**a**) SEM image of the material sampled by the 0.25 μm cut-off filter with a magnification of 50 μm, (**b**) EDX elemental analysis, (**c**) contribution of carbon and oxygen is dominant

**Fig. 7:**
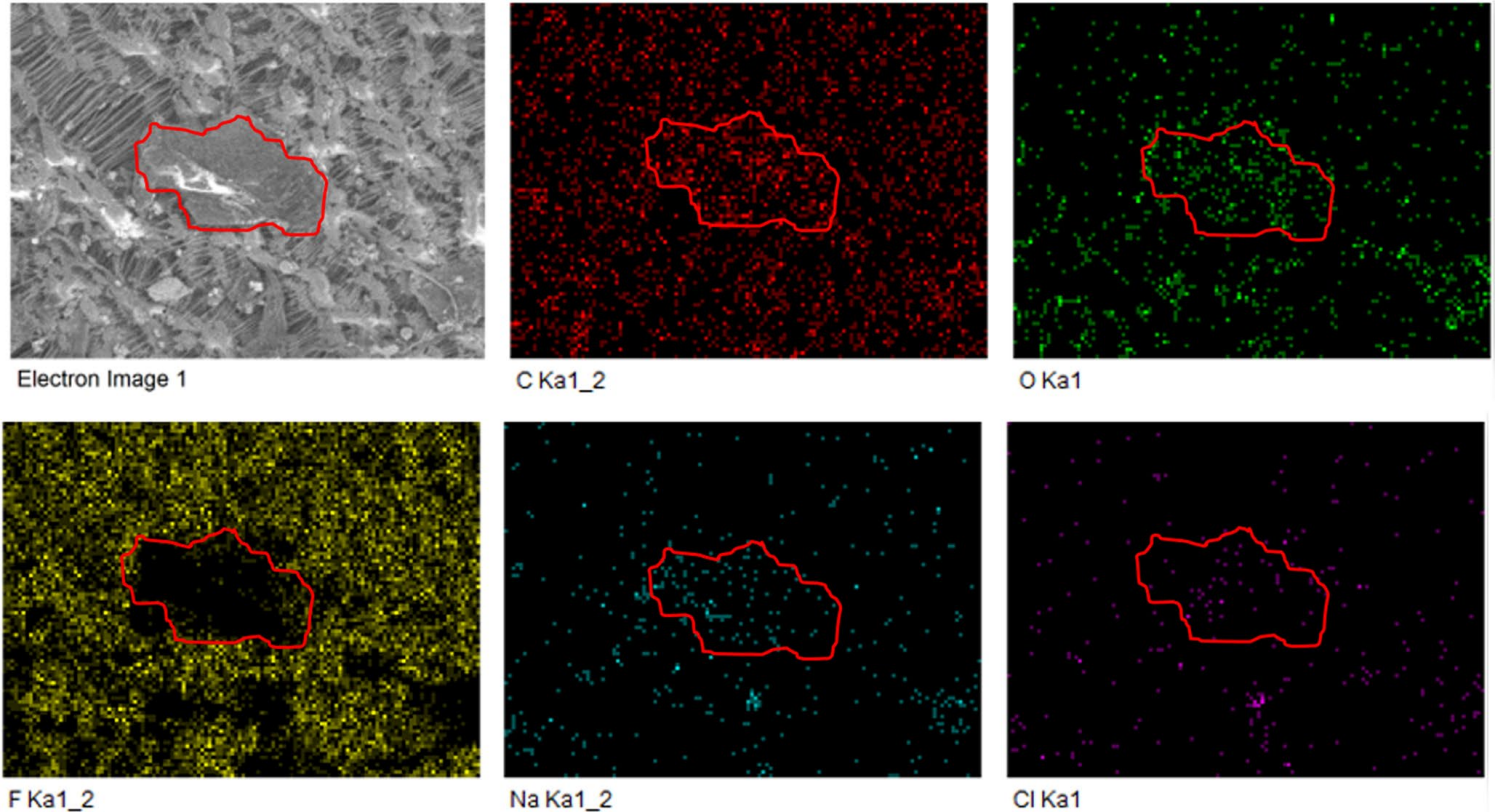
Elemental composition of the filter sample of 2.5 μm, showing a particle made of carbon and oxygen

The morphological study by SEM (Fig. 6) determined the presence of irregular carbon-based particles with a tendency to resemble a bent leaf shape. To verify that the carbon detected is coming from the GO present in the filament, a raw piece of the black filament was analysed before and after printing (Fig. SI1-2). Elemental composition (EDX) identified particles containing carbon and oxygen in a percentage compatible with GO in the 0.25 μm and 2.5 μm (Figs. 6 and 7), 0.5 μm and 1 μm samples (see Supplementary Material, Fig. SI3-6).

SEM/EDX results are not presented as definitive proof of GO, as Carbon and Oxygen alone cannot confirm its presence, since they can be found in any component of the sample (PTFE filter, PLA). However, the observed morphology is fully compatible with GO according to common characterization techniques, conforming supportive evidence. The following Raman spectroscopy analysis provides more reliable confirmation of GO.

#### Raman spectroscopy

Raman spectroscopy was performed in four types of samples to assess the presence and transfer of the GO through the printing process. The original PLA + GO black thread was measured to verify the presence of GO in the filament and compared with a printed strip from the same filament to determine if the GO structure was preserved after melting and extrusion. This was compared with the aerosol collected on the 0.25 and 1 μm PTFE filters from the Sioutas impactor. As a control, a white filament without GO was analysed to confirm that the detected GO-related bands are from the nanostructured filament.

**Fig. 8:**
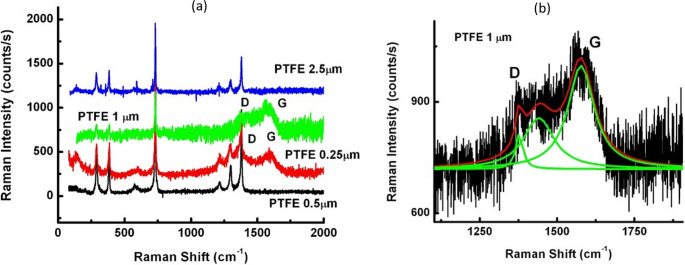
(**a**) Raman spectra of samples collected with the PTFE filters of Sioutas, (**b**) Lorentzian function fitting to the Raman spectrum corresponding to the 1 μm sample filter

The vibration bands of the PTFE filter [57] are observed in all samples (Table SI1). In the 1 μm and 0.25 μm PTFE filter, collected particles showed the D and G bands of GO (Fig. 8(b)) [58]. The green line fits with the Lorentzian functions, whereas the red line is the sum of the Lorentzian functions, and the black line is the experimental spectrum.

Comparing the results of Fig. 8(b) and Table 5(a) with those obtained in the literature, the line shape of the D- and G-bands and the fitting values obtained from the full widths at half height (FWHM) correspond to disordered carbon or GO [59]. The vibrational band at 1450 cm^–1^ corresponds to the CH_2_, CH_3_ deformation of the PTFE polymer.

**Table 5:** Summarised band position and FWHM corresponding to the Lorentzian fitting for reference spectrum, PTFE-collected aerosol, and PLA + GO printed samples

Fitting parameters of the Lorentzian functions of the Raman spectrum	Assignment	Peak position (cm^–1^)	FWHM (cm^–1^)
(a) Band position and FWHM corresponding to the Lorentzian fitting	D-band	1378.3	27.3
	CH_2_, CH_3_	1458.0	119.6
	G-band	1578.0	103.1
(b) Sample collected with the 0.25 μm stage PTFE filter	CF_2_ symmetric stretching	1200.0	37.1
	CF_2_ asymmetric stretching	1294.7	19.4
	D-band	1354.9	198.4
	NO_3_^–^	1373.0	11.3
	G-band	1587.4	120.1
(g) Raw black thread (PLA + GO filament before printing)	PLA	1299.6	31.5
	D-band	1358.0	37.6
	PLA	1387.2	8.0
	CH3	1457.0	19.7
	G-band	1581.0	16.4
(l) Printed black thread (PLA + GO)	PLA	1297.9	17.0
	D-band	1346.0	49.8
	PLA	1387.0	14.4
	CH_3_	1457.0	18.2
	PLA	1547.2	7.9
	G-band	1579.0	15.2

**Fig. 9:**
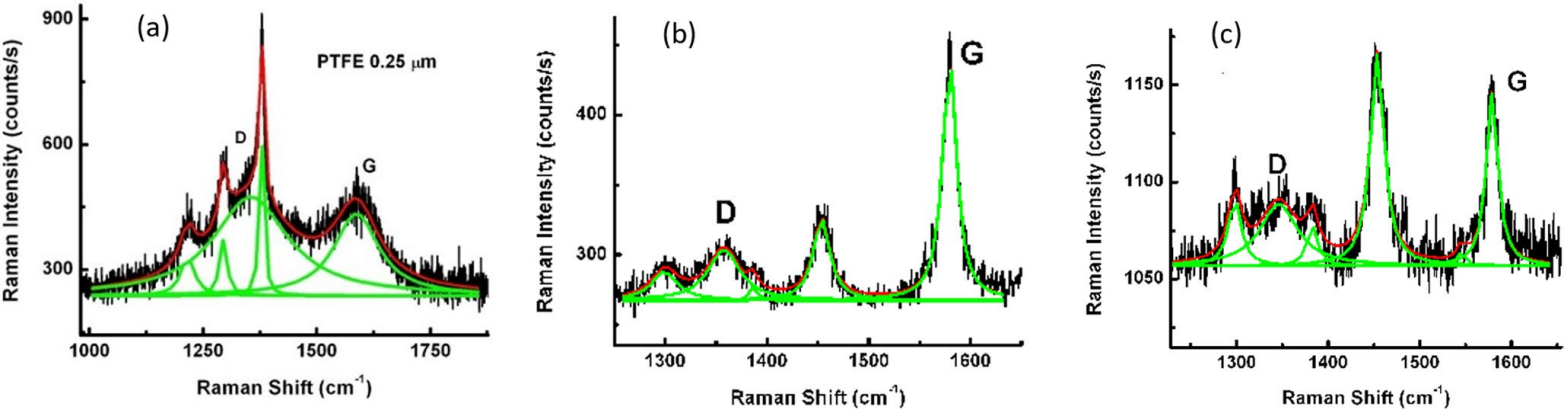
(**a**) Raman spectrum of the sample collected with the 0.25 μm PTFE filter in the spectral region of GO, (**b**) Raman spectrum of raw black thread in the spectral region of GO, (**c**) Raman spectrum of the printed black strip in the spectral region of PLA + GO

In Fig. 9(a), the D- and G-bands of the disordered carbon/GO [59] of the sample collected with the 0.25 mm PTFE filter are clearly observed. In addition, different vibrational bands corresponding to the PTFE filter also appear in the same spectral region. Figure 9(b) corresponds to the Raman spectrum of the raw black thread (PLA + GO). The D- and G-bands together with the vibrational bands of PLA polymer are clearly observed. Table 5(b) and (c) also collects the fitting parameters of the Raman spectra corresponding to the peaks of Figs. 9(a) and (b) respectively.

In the raw black thread sample, the values of the vibrational frequencies of the D- and G-bands, as well as the FWHM are consistent to the values observed for GO layers [58]. Figure 9(c) shows the Raman spectrum of the printed black strip. In this case the D-and G-bands of GO as well as vibrational bands corresponding to the PLA polymer are clearly visible. Table 5(d) also collects the fitting parameters of the Raman spectra corresponding to the peaks of Fig. 9(c).

The values of the fitting parameter collected in Table 5(d) coincide with the values of the GO layers. As a control, the spectrum of the white thread sample was also measured. As expected, this spectrum does not show bands corresponding to graphene or GO layers. In the white filament, made solely by PLA, no carbon traces were detected, while in the original black filament, made of PLA + GO, it was confirmed the presence of GO. Therefore, the GO found comes exclusively from the GO added to the filament. Besides, the white filament shows a clear difference between the Raman spectrum of the PLA and the PTFE of the filter, which significantly reduces the risk of confusing the two compounds during their study. In summary, in the samples collected with the 1 μm and 0.25 μm PTFE filters it was confirmed the presence of GO.

### Statistical analysis

As previous measurements show, PNC is lower than the proposed limits 40,000 pt/cm^3^ [60, 61] and decreasing with time, posing low risk. Therefore, following the decision criteria of UNE-EN 17058:2018, further analysis must be taken.

A second printing process was carried out. The PNC was measured in 15-minute periods for both, the NF and FF, gathering 12 recordings, exceeding the 10 required by the standard, using the same devices described previously.

Correlations between NF and FF, and implications between background and activity were analysed. After assessing the stationarity of the data by a first derivative, the rules for the basic ARIMA assessment were applied.

**Fig. 10:**
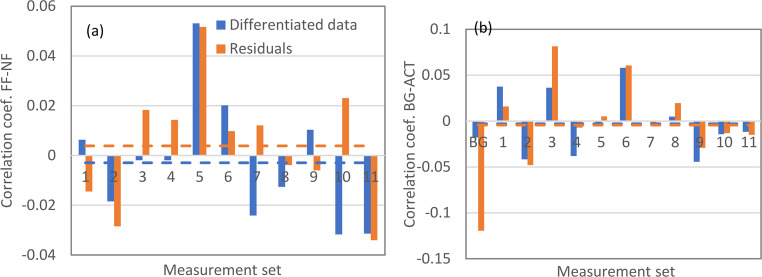
(**a**) Pearson’s product moment correlation coefficient for the relationship between NF and (**b**) FF and BG and activity

The Pearson’s product moment correlation coefficient measures the strength and direction of a linear relationship between two sets. In both cases, if there were an effect of the activity, it would be expected to obtain a positive correlation, meaning that, as one variable increases, the other tends to increase, reaching 1 to a perfect positive correlation. However, it can be seen in Fig. 10 that is positive in some cases and negative in others, with an average of − 0.003 for the NF-FF relation (Fig. 10(a)), and for the background – activity (Fig. 10(b)), therefore, correlation can be discarded.

The same occurs for the correlation of the residuals of applying the ARIMA model, searching the correlation on the differences between the observed values and the values predicted. It provides insights into whether there are patterns or relationships not captured by the models. If the residuals of two models are correlated, it may indicate that there are common factors influencing both models, suggesting that some information is not accounted for by each model individually. But as previously, values are fluctuating around zero, with averages of 0.0039 for the NF-FF relationship and − 0.0043 for the background-activity.

**Fig. 11:**
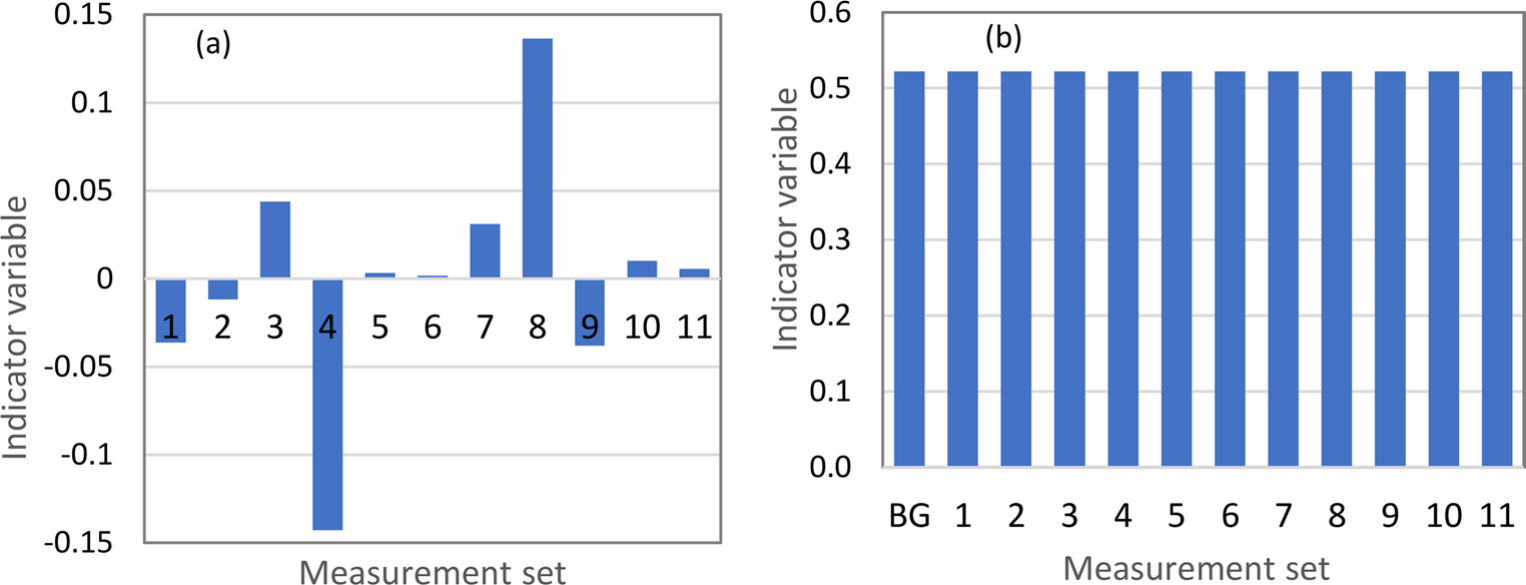
(**a**) Indicator variable of the relationship between NF-FF and (**b**) BG-activity

The coefficients for the exogenous variables in the ARIMA model indicate the expected change in the dependent variable for a one-unit change in the corresponding independent variable, assuming all other variables are held constant. Thus, for the case of changes in the FF due to modifications in the NF, it can be seen in Fig. 11(a) that the coefficients have small values and deviate between measurement sets. This suggests no detectable linear relationship within the limits of the current dataset, although nonlinear or lagged effects cannot be excluded. Accordingly, the results should be interpreted as indicative rather than conclusive.

The relationship of the background versus the activity on the other hand, is constant with a value of 0.52 (Fig. 11(b)). This indicates that a one-unit increase in the indicator variable is associated with a 0.5-unit increase in the dependent variable, all else being equal.

**Fig. 12:**
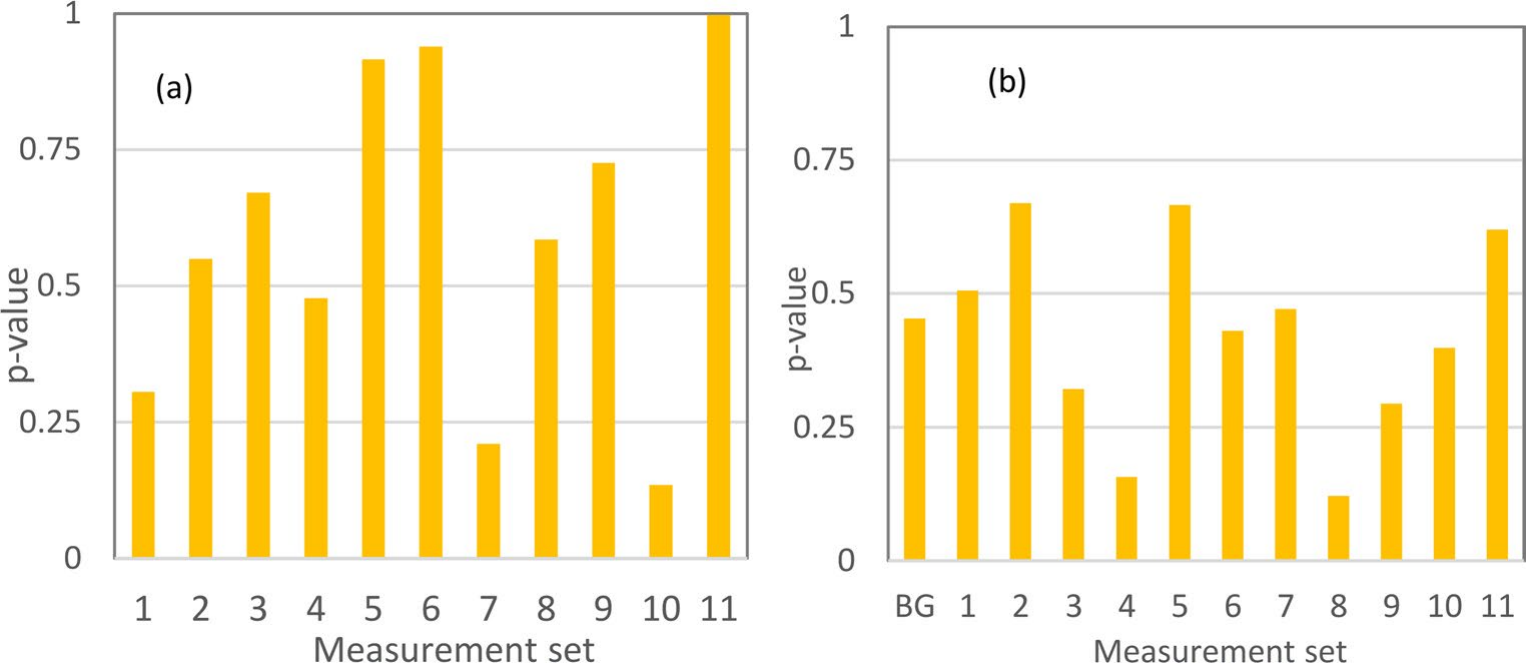
(**a**) p-values from the Granger causality test for the relations NF-FF and (**b**) BG-activity

Since the correlation of residuals alone does not necessarily imply causation, the Granger causality test was also applied. In Fig. 12, it is summarized the *p*-values obtained, well above the significance level (*p* < 0.05), therefore accepting the null hypothesis and concluding that Granger causality does not exist in any case, i.e., there is no relation between the slopes in FF-NF or Background - Activity.

## Discussion

The nanoparticles measured showed low PNCs, with averages of 2.10 × 10^3^ pt/cm³ with CPC at NF and 2.75 × 10^3^ pt/cm³ with P-Track at FF. This difference in concentrations might be due to agglomerates formed near the emission source, which are weakly bounded and can partially fragment and disperse as they cool. There is also a continuous small decrease during the whole 3D printing process. This behaviour is consistent with previous reports for PLA-only filaments [16, 62]. This may be attributed to agglomeration and aggregation phenomena, which reduce the number of detectable particles over time, as it was observed on the sample characterized by microscopy, as well as for the low extrusion temperature used. In any case, further repetitions and dedicated instrumentations would confirm these dynamic processes and enlighten the PNC differences. Spinazeé et al. [33] proposed a reference value of 8-h TWA 40,000 pt/cm^3^ according with the general proposal of NIOSH [33, 63–65]. The PNC averages measured in both NF and FF are below one tenth this reference value.

Given that VOCs were measured once (at the end of the first printed process), and according to EN 689:2019 + AC:2019 standard [64], it is not possible to make a compliance decision. However, as measurements were taken under the worst-case scenario, it provides a reliable indication of the maximum potential workplace exposure to the identified VOCs. With the standard measurements methods and analysis employed, only formaldehyde was detected (lowest value range) amongst the potentially hazardous chemicals released during FDM with the PLA + GO filament. Being a carcinogenic agent, Directive 2004/37/CE [65] should be applied. In any case, all concentrations were below the proposed limits.

From the direct measurement results, it can hence be assumed that there are two particle sources: on the one hand, micrometre-sized particles with relatively small surface area, and on the other hand, sparse particles with high surface area but low volume, likely representing volatilised PLA during printing and the released GO particles, respectively.

In the scenario studied, the generic decision logic method and ARIMA model were applied to evaluate source contribution. The results point out that NF emissions do not significantly affect FF concentrations, and no secondary background sources were detected at FF. However, although the overall nanoparticle concentrations are low, SEM and Raman analyses indicated the presence of GO within the respirable aerosol, with a chemical composition identical to the printed material, verifying that the carbon detected is coming exclusively from the embedded GO by comparison with the analysis of a raw black PLA + GO filament before and after printing, as well as with a reference sample of PLA without graphene. Therefore, emissions cannot be considered negligible: exposure is limited in magnitude but not absent, and the presence of GO-containing particles should still be taken with caution.

Previous studies reporting particle emissions from PLA-based filaments [10, 13, 26] focused only on particle number or nano-size distribution range and, to our knowledge, did not confirm if there was an embedded nanomaterial actually being released into the airborne fraction. In this sense, the presented results are consistent in terms of emission magnitude with those studies, but provide an additional contribution by confirming, through SEM and Raman the transfer of GO from the PLA + GO filament to the respirable aerosol. As a result, they cannot eliminate the possibility of exposure of workers to the NOAAs added to the PLA filament [23].

## Conclusions

This pilot study in a real 3D printing scenario with PLA + GO filament demonstrates the applicability of the tiered standardized protocols proposed in EN 17058:2018 for assessing exposure to nanomaterials.

To ensure the data quality, a second printing session with 12 measurements were successfully checked with ARIMA. The results confirmed minimal propagation of the released particles from NF to FF, and no significant effect of the activity versus the background, rejecting potential secondary sources.

Nevertheless, the detection of GO, even at low levels, justifies the implementation of preventive exposure control measures under Tier 2 of EN 17058:2018, ensuring that there is no particle propagation, However, according to EN 17058:2018, Sect. 6.3.3.3, when Tier 2 analysis does not fully exclude exposure, a Tier 3 comprehensive assessment is generally required to further quantify personal exposure, although alternatively, the assessor may choose to implement risk management measures to mitigate potential exposure.

While scanning electron microscopy (SEM) identified microparticles (~ 2.5 μm), it was insufficient alone to confirm GO presence. Raman spectroscopy proved to be a valuable tool for the reliable identification of carbon-based nanomaterials and allotropes [66], complementing the SEM/EDX information and supporting more accurate risk evaluation and preventive management. By combining near-/far-field real-time monitoring with SEM and Raman spectroscopy, it was confirmed that embedded GO particles can be released from the filament into the respirable aerosol, meaning that exposure is not limited to the inherent process- generated nanoparticles.

Overall, the monitoring approach presented here supports proactive exposure assessment and risk management, contributing to safer adoption of 3D printing technologies using engineered nanomaterials, but also raising new questions that require further comprehensive studies including Tier 3 assessments, repeated sampling, and evaluation under diverse workplace conditions.

## Supplementary Information

Below is the link to the electronic supplementary material.

Supplementary Material 1 (DOCX 3.36 MB)

## Data Availability

The data that support the findings of this study are openly available in “Mendeley Data” [67].
