# Supplementary material for: Monitoring and risk assessment of airborne graphene oxide released during a pilot study of 3D printing with polylactic acid filaments
Source: J Environ Health Sci Eng. 2026 Aug 7;24(2):33. doi: 10.1007/s40201-026-00998-w (PMC13451170; doi:10.1007/s40201-026-00998-w)
Supplement: Supplementary file 1 [file 40201_2026_998_MOESM1_ESM.docx]

**Monitoring and Risk Assessment of Aerosolized Graphene Oxide Released During 3D Printing with PLA-Based Filaments**

Ciro Salcines^1^, Maidá Domat^2^, Javier Ruiz^3^, Laura Castanon-Jano^4^, Elena Blanco-Fernandez^5^, Rafael Valiente^6^

^1^Health and Safety Unit, Universidad de Cantabria, 39005 Santander, Spain. ([0000-0003-2600-3552](https://orcid.org/0000-0003-2600-3552))

^2^Area of Applied Physics, Universidad de Oviedo, 33007 Oviedo, Spain. ([0000-0002-0526-5425](https://orcid.org/0000-0002-0526-5425))

^3^Area of Health and Safety, FREMAP - Mutua Colaboradora con la Seguridad Social nº 61, 28222 Majadahonda, Spain.

^4^Area of Manufacturing Processes, Universidad de Cantabria, 39005 Santander, Spain. ([0000-0002-5968-2726](https://orcid.org/0000-0002-5968-2726))

^5^GITECO Research Group, Universidad de Cantabria. 39005 Santander, Spain. ([0000-0002-7010-2649](https://orcid.org/0000-0002-7010-2649))

^6^Applied Physics Department, Universidad de Cantabria, 39005 Santander, Spain. ([0000-0001-9855-8309](https://orcid.org/0000-0001-9855-8309))

*Corresponding author email*: domatmaida@uniovi.es

**Supplementary Material**

Some data are presented here to complement and expand on the information presented.

**Table S1** PTFE Polymer vibration frequencies

(a)

(b)

**Fig. S1** (a) Raw piece of the black filament (PLA+GO) before being printed, (b) oxygen and carbon appear at almost same ratios of 50%

(a) (b)

**Fig S2** (a) Raw piece of the black filament (PLA+GO) after being printed, (b) oxygen and carbon appear at almost same ratios of 50%

SI3(a)

(b)

(c)

**Fig. S3** (a) SEM image of material collected by the 0.5 µm PTFE filter, (b) EDX with the elemental analysis of the filter and sample, and (c) percentages of different elements obtained with the EDX analysis

**Fig. S4** Elemental composition of the filter sample of 1 µm, showing a particle made of carbon and oxygen

**Fig. S5** (a) SEM image of material collected by the 1 µm PTFE filter, (b) EDX with the elemental analysis of the filter and sample, and (c) percentages of different elements obtained with the EDX analysis

16(b)

SI5(a)

(b)

(c)
